# Impact on time to active antimicrobial therapy with 24-hour pharmacist review of Accelerate Pheno BC Kit results

**DOI:** 10.1017/ash.2022.274

**Published:** 2022-08-05

**Authors:** Patrick M. Kinn, Bradley Ford, Kelly M. Percival, Lukasz Weiner, Dilek Ince

**Affiliations:** 1 Department of Pharmaceutical Care, University of Iowa Hospitals and Clinics, Iowa City, Iowa; 2 Department of Pathology, University of Iowa Hospitals and Clinics, Iowa City, Iowa; 3 Division of Pediatric Infectious Diseases, Department of Pediatrics, University of Iowa Hospitals and Clinics, Iowa City, Iowa; 4 Division of Infectious Diseases, Department of Internal Medicine, University of Iowa Hospitals and Clinics, Iowa City, Iowa

## Abstract

The Accelerate Pheno platform provides rapid identification and susceptibility data. We demonstrate successful incorporation of 24-hour pharmacist review of Accelerate Pheno results that reduced the number of patients going >3 hours from result without an order for active antimicrobial therapy from 29 (2.8%) of 1,043 to 9 (0.85%) of 1,053 (*P* < .001).

Bloodstream infections are associated with significant morbidity and mortality, particularly with delays in appropriate antibiotic therapy.^1^ Rapid pathogen identification and susceptibility testing can improve outcomes, especially if implemented with an antimicrobial stewardship program (ASP) intervention.^2–4^

The Accelerate Pheno system (Accelerated Diagnostics, Tucson, AZ) is a fully automated platform that uses an array of probes to provide identification of common bacterial pathogens from a positive blood culture and subsequent phenotypic antimicrobial susceptibility testing (AST). This system can provide identification and AST results within 2 and 7 hours of identifying a positive blood culture, respectively.^5^

Our clinical microbiology laboratory implemented Accelerate Pheno in September 2018 with real-time intervention by the ASP during standard workday hours, leading to decreased time to optimal therapy.^6,7^ We identified an opportunity to further improve patient care by reducing delays in initiation of active antimicrobial therapy outside regular ASP work hours. In this study, we evaluated the impact of incorporating 24-hour unit-based pharmacist review of rapid blood-culture results.

## Methods

This single-center, quasi-experimental study was conducted at an 811-bed academic medical center that includes a children’s hospital. Initial implementation of Accelerate Pheno (cf, the preimplementation cohort) included distribution of results to the primary medical team at the time of organism identification and again when preliminary rapid susceptibilities became available. This data distribution was paired with an alert by page to the ASP Monday through Friday 07:00–17:00; after-hours results were reviewed the next business day.

Distribution of real-time results was broadened in the postimplementation cohort to include unit-based pharmacists in the evenings (17:00–06:59) and all day on weekends, with the expectation to intervene only if an escalation in therapy was needed. Case-based education on culture interpretation was provided to all unit-based pharmacists prior to implementation along with a summary “cheat” sheet providing preferred and acceptable antimicrobial options based on the organism identified and morphology on Gram stain.

We compared active therapy initiation during an ASP-only review of Accelerate Pheno results from September 2018 to October 2019 (cf, preimplementation cohort) compared to ASP plus 24-hour pharmacist review from December 2019 to January 2021 (cf, postimplementation cohort). The primary outcome was delayed antimicrobial initiation defined as not having an active therapy ordered within 3 hours of Accelerate Pheno result availability. Active therapy was defined as a presumably active antimicrobial as outlined by the summary sheet and antibiogram when only organism identification was available and as a confirmed active antimicrobial when susceptibilities subsequently became available. A maximum of 1 delayed antimicrobial initiation per sample contributed to the primary outcome. A secondary outcome was the number of unit-based pharmacist interventions, with a maximum of 1 per patient. All patients who were admitted to the hospital at the time of the Accelerate Pheno result and whose result was not deemed a contaminant were included. Data were abstracted via chart review, and the institutional review board approved the study. Statistical analyses were conducted using the Fisher exact test.

## Results

In total, 2,909 positive blood-culture results were included in the initial data collection. Among them, 813 were determined to be likely contaminants, resulting in 2,096 unique episodes of bloodstream infections: 1,043 in the preimplementation cohort and 1,053 in the postimplementation cohort.

Table 1 describes characteristics of these infections, including the patient unit at time of culture positivity and organism type identified by Accelerate Pheno. Organisms under the “unidentified/off panel” category reflect isolates that were not identified by the Accelerate Pheno system. This category represents isolates that were truly off panel and isolates that were on the panel but the Accelerate Pheno system failed to identify. Ultimately, these were identified by alternative techniques. The most common organism types in this category were *Candida* spp (n = 55), *Bacteroides fragilis* (n = 35), *Escherichia coli* (n = 32), and *Enterococcus faecalis* (n = 25).

**Table 1.** Description of Positive Blood-Culture Samples

Variable	Preimplementation Cohort (n = 1,043), No. (%)	Postimplementation Cohort (n = 1,053), No. (%)
**Unit type of positive culture**		
Adult ICU	307 (29)	337 (32)
Adult ward	593 (57)	561 (53)
Pediatric ICU	53 (5.1)	67 (6.4)
Pediatric ward	73 (7.0)	74 (7.0)
Emergency department	17 (1.6)	14 (1.3)
**AP organism identification**		
Enterobacterales^a^	284 (27)	276 (26)
*Staphylococcus aureus*	265 (25)	254 (24)
Unidentified/off panel	179 (17)	211 (20)
*Streptococcus* spp	103 (9.9)	84 (8.0)
*Enterococcus* spp	76 (7.3)	76 (7.2)
Coagulase-negative *Staphylococcus* spp	65 (6.2)	75 (7.1)
*Pseudomonas aeruginosa*	42 (4.0)	41 (3.9)
*Candida* spp	15 (1.4)	17 (1.6)
Polymicrobial	11 (1.0	19 (1.8)
*Acinetobacter baumannii*	3 (0.3)	0 (0.0)
**Delayed antimicrobial initiations by organism type**		
Enterobacterales	9 (0.9)	2 (0.2)
*Staphylococcus aureus*	8 (0.8)	1 (0.1)
*Enterococcus* spp	5 (0.5)	1 (0.1)
*Pseudomonas aeruginosa*	4 (0.4)	0 (0.0)
Unidentified/off panel^b^	1 (0.1)	4 (0.4)
*Candida* spp	1 (0.1)	1 (0.1)
Polymicrobial	1 (0.1)	0 (0.0)

Note. ICU, intensive care unit; AP, Accelerate Pheno system.

a Enterobacterales group includes Accelerate identification of *Citrobacter* spp, *E. coli*, *Enterobacter* spp, *Klebsiella* spp, *Proteus* spp, and *Serriata marcescens*.

b Unidentified/off-panel organisms that were counted as delayed starts were all unidentified by rapid diagnostic but had Gram-stain morphology consistent with budding yeast and ultimately identified as *Candida* spp.

**Fig. 1.**
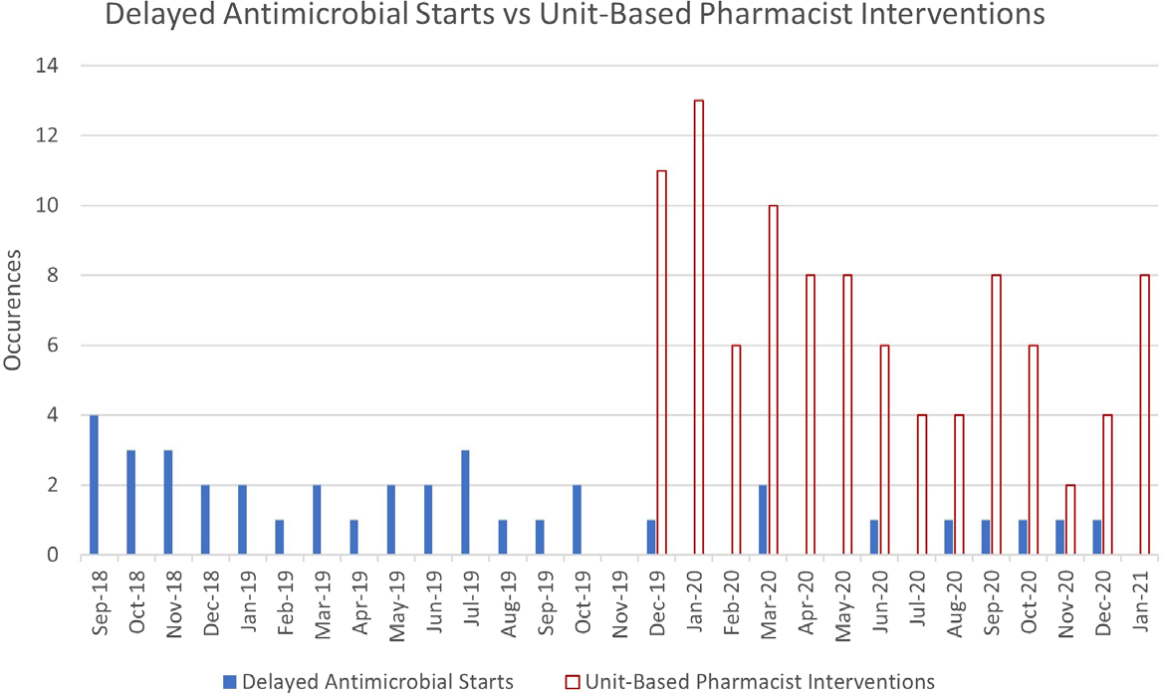
Rate of pharmacist intervention and delayed antimicrobial starts. The figure displays the rates of primary and secondary outcomes over time. The primary outcome (blue filled bars) was delayed antimicrobial initiation defined as not having an active therapy ordered within 3 hours of Accelerate Pheno result availability. Active therapy was defined as a presumably active antimicrobial as outlined by an institutional summary “cheat” sheet and antibiogram when only organism identification was available and then as a confirmed active antimicrobial when susceptibilities subsequently became available. Secondary outcome (red unfilled bars) was unit-based pharmacist intervention for antimicrobial adjustment. Note: implementation of 24-hour pharmacist review began in November 2019. No data were included from that transitionary month.

Figure 1 describes the number of delayed antimicrobial starts by month. In the preimplementation cohort, 29 delayed antibiotic starts occurred, representing 2.8% of bacteremia episodes. In the postimplementation cohort, 9 delayed antibiotic starts occurred, representing 0.85% of bacteremia episodes (*P* < .001). Unit-based pharmacists intervened in 98 episodes over 14 months to help escalate antibiotic therapy to include an active agent, with an average of 7 interventions per month.

## Discussion

In this study, we evaluated the impact of the addition of 24-hour pharmacist review of positive blood-culture results run on the Accelerate Pheno system at our institution. In the absence of adequate resources for 24–7 ASP intervention in real time, patient safety concerns, such as escalation of therapy to ensure active antibiotic therapy, needed to take precedence outside regular work hours. Our results suggest that unit-based pharmacists may be effective in ensuring active therapy for patients with bacteremia.

The initial implementation of Accelerate Pheno reinforced the value of pairing rapid diagnostic techniques with ASP review to decrease time to optimal therapy, especially in the absence of prior rapid diagnostics for bloodstream infections.^2–4^ The rapid identification of most bloodstream pathogens via Accelerate Pheno (81.4% in our analysis) required education and adjustments to previous routines. As was the experience at our institution, previous studies have demonstrated that implementation of the Accelerate Pheno system improves time to optimal antimicrobial therapy, particularly when paired with ASP review.^6–8^ However, an opportunity remained to ensure that patients were at least on an active antimicrobial based on the results of the Accelerate Pheno system in the hours that the ASP team was not in house to review results in real time.

This study demonstrated that incorporation of 24-hour pharmacist review was associated with a significant reduction in the number of patients with delay of active antimicrobial therapy beyond 3 hours of result availability via Accelerate Pheno. Pharmacists were trained to assess the results of the blood-culture diagnostic and to intervene as needed through direct communication with the treating provider when an opportunity to escalate antimicrobial therapy (based on the identified pathogen and/or rapid antimicrobial susceptibilities) was identified. Unit-based pharmacists reviewed hundreds of blood-culture results over a period of 14 months and intervened to escalate therapy in 98 patients. Although this intervention added a new responsibility to the unit-based pharmacist staff and thus increased workload, it successfully resulted in a decrease in the number of patients inadequately covered for bloodstream pathogens from 29 (2.8%) in the preimplementation cohort to 9 (0.85%) in the postimplementation cohort. Other studies have similarly concluded that pharmacist incorporation in assessment of blood-culture results can be helpful; however, this study is the first to focus on improving delayed initiation of active antimicrobial therapy with the Accelerate Pheno system through a pharmacist-led mechanism.^9,10^

This study had several limitations. We identified the potential for maturation effect secondary to the stewardship team making recommendations during standard workday hours prior to the postimplementation phase (and during it) as well as the potential for unmeasured confounders. Additionally, the primary outcome measured in this study (delayed antimicrobial therapy beyond 3 hours of positive Accelerate Pheno result) is not necessarily directly associated with clinical outcomes such as morbidity and mortality. Although this quality improvement intervention did have the desired effect of improving time to escalation in therapy for uncovered bloodstream pathogens, delays in antimicrobial therapy remained. Continued review of all positive blood-culture results by the ASP (whether in real-time or retrospectively) helped identify opportunities for continued education to healthcare providers of all types including pharmacists, advanced practice providers, learners, and attending physicians. It also helped drive process improvements throughout the hospital often in collaboration with the clinical microbiology laboratory.

## Supplementary material

For supplementary material accompanying this paper visit https://doi.org/10.1017/ash.2022.274. click here to view supplementary material
