# Supplementary material for: Impact on time to active antimicrobial therapy with 24-hour pharmacist review of Accelerate Pheno BC Kit results
Source: Antimicrob Steward Healthc Epidemiol. 2022 Aug 5;2(1):e132. doi: 10.1017/ash.2022.274 (PMC9726536; doi:10.1017/ash.2022.274)
Supplement: Supplementary file 1 [file S2732494X22002741sup001.docx]

**Supplemental Document: Expanded Methods**

**Microbiology Lab Processes:** Microbiology laboratory processes were consistent throughout the pre- and post-implementation cohorts with the only change being inclusion of automated distribution of results to the unit-based pharmacists in the off-hours.

Blood culture bottles were incubated on-site in the microbiology laboratory in the Bactec FX (BD) system. They were monitored continuously for growth and laboratory staff were available 24 hours a day for review of positive results and entry into the electronic health record (EHR). Laboratory staff called Gram stain results to nursing staff regardless of the time of day as a critical result if the result was off-panel or unidentified by AP. Otherwise, call was made when AP ID became available. Laboratory staff distributed results from AP organism identification and rapid antimicrobial susceptibility results as critical results regardless of the time of day to the floor nursing staff who subsequently notified primary team as per protocol.

Organism identification was confirmed with MALDI-TOF MS (Druker Daltronics) regardless of whether identification of sample was provided by AP. Antimicrobial susceptibility testing was conducted per laboratory protocol utilizing Vitek 2 (*bioMerieux)* or ThermoFisher Sensitite (Thermo Scientific) panels utilizing Clinical and Laboratory Standards Institute (CLSI) breakpoints.

**Antimicrobial Stewardship Program Review of Results:** Distribution of microbiology results to the antimicrobial stewardship program were consistent throughout the pre- and post-implementation cohorts.

An automated page was distributed to the ASP pager upon entry of updated blood culture results by the microbiology laboratory technician into the EHR. This page was distributed to the ASP pager if the result arrived between the hours of 0700 and 1700 on Monday through Friday. Pager may be reviewed by any member of the antimicrobial stewardship team but is most commonly reviewed by pharmacist members of the team. It also populated an Epic in-basket pool for review by the ASP that could be reviewed retrospectively if new results occurred in the off-hours.

**Unit-Based Pharmacist Review of Results:** The Department of Pharmaceutical Care provides clinical pharmacy services for all patients admitted to the University of Iowa Hospitals and Clinics. The acute care division operates with a mix of operationally focused pharmacists that provide product verification and distribution and patient care unit-based clinical pharmacists (referred to as unit-based pharmacists) that provide order verification and a variety of clinical services. During a typical day-time shift, each patient care unit is serviced by one or two unit-based pharmacists. These shifts are consolidated to a total of 7 unit-based pharmacist coverage teams during the evenings and weekends and down to a total of 3 pharmacists during the overnight period. These shifts are staffed by clinical pharmacists and pharmacy residents.

All unit-based clinical pharmacists and pharmacy residents underwent required training sessions conducted by the ASP. Sessions included the following information: background on the AP system, discussion of the system for distribution of AP results, review of basic microbiological pathogens, overview of the AP cheat sheet, case-based learning exercises, and outline of expectations of unit-based pharmacist staff. Sessions were available in-person and were followed by competency tests conducted through a web-based platform. Sessions are provided for all new hires including pharmacy residents. Competencies are completed at time of new-hire orientation and annually for all unit-based pharmacists and pharmacy residents.

In the post-implementation cohort, AP results were distributed to unit-based pharmacists when resulted outside of standard ASP hours (0700-1700 Monday through Friday). An automated page was distributed to a pager within the central pharmacy which is monitored by a pharmacist. Result could be assessed by that pharmacist independently if responsible for the care of the corresponding AP result or they could triage the result to unit-based pharmacist responsible for that patient.
